# School-Based Prevention Targeting Non-Suicidal Self-injury: A Pilot Study

**DOI:** 10.3389/fpsyt.2020.00437

**Published:** 2020-05-29

**Authors:** Imke Baetens, Christine Decruy, Shokoufeh Vatandoost, Birgit Vanderhaegen, Glenn Kiekens

**Affiliations:** ^1^Brussels University Consultation Center, Department of Psychology, Faculty of Psychology & Educational Sciences, Vrije Universiteit Brussel, Brussels, Belgium; ^2^Department of Neuroscience, School of Medicine, Institute for Family and Sexuality Studies KU Leuven, Leuven, Belgium; ^3^Department of Neurosciences, Center for Public Health Psychiatry KU Leuven, Leuven, Belgium

**Keywords:** non-suicidal self-injury, prevention, school-based, general well-being, psychoeducation

## Abstract

Non-suicidal self-injury (NSSI) is prevalent in adolescence and is associated with increased risk for a variety of subsequent negative mental health outcomes, necessitating an evidence-based preventive approach. This pilot study examines the potential iatrogenic effects and feasibility of an evidence-based school program for the prevention of NSSI. Differences are examined between a general in-classroom prevention program (Happyles) and this program combined with a 1-h in-classroom psychoeducation module on NSSI (HappylesPLUS) in terms of primary (e.g., delay in NSSI onset and decrease in NSSI frequency, urges, probability of future engagement) and secondary outcomes (e.g., psychological distress, emotion regulation, help-seeking, and stigma) using a mixed-method design. A total of 651 secundary school pupils (M_age_ = 12.85 years; 49.8% female versus 50.2% male) were assigned to the Happyles program and HappylesPLUS. Participants filled out validated self-report questionnaires pre (T0) and post (T1, 6 weeks after T0) the school prevention program, including the Youth Outcome Questionnaire (YOQ), the Brief Non-Suicidal Self-Injury Assessment Test (BNSSI-AT), the Difficulties in Emotion Regulation Scale (DERS), the Attitudes Toward Seeking Professional Psychological Help Scale—Short Form (ATSPPH-SF), and the Peer Mental Health Stigmatization Scale (PMHSS). Qualitative semi-structured interviews (at T2,6 weeks after T1) were conducted with participants with and without a history of NSSI. Overall, results show no iatrogenic effects of the NSSI-focused psychoeducation module. In terms of our primary outcome, both groups reported a reduced likelihood of future NSSI engagement from T0 to T1. Regarding secondary outcome measures, we also observed increased emotional awareness in both groups. The qualitative data suggest that the addition of the NSSI-specific module to the Happyles program may have direct benefits to some students with lived experience, such as increased help-seeking behavior for NSSI. Findings of this pilot study show that incorporating NSSI-specific modules into evidence-based school prevention programs is feasible and does not lead to iatrogenic effects. Future work is needed to evaluate the potential (longer-term) benefits of incorporating NSSI-focused modules to evidence-based mental health programs in the prevention of NSSI.

## Introduction

Non-suicidal self-injury (NSSI) is the deliberate self-inflicted damage of one's own body tissue without suicidal intent (1) and includes behaviors such as cutting, burning, and hitting oneself (2). The lifetime prevalence of NSSI is around 17.2% among adolescents (3), with 10% reporting a 12-month prevalence of NSSI (2) and 6% meeting criteria for the recently proposed Diagnostic and Statistical Manual of Mental Disorders (DSM-5) NSSI disorder (4). Adolescence carries the highest risk for onset of NSSI, with prevalence rates starting to increase in early adolescence, between the ages of 11 and 13 years (e.g., 5, 6), and reaching a peak in mid-adolescence between the ages of 14–15 years (5, 6).

For most adolescents, NSSI is a way to cope with intense emotions, self-critical thoughts, or to signal distress to others (2). NSSI is prospectively associated with increased risk for a variety of negative mental health outcomes, including anxiety, depression, disordered eating, and personality disorders (7–10). For example, an earlier age of onset of NSSI (before age 13) and longer duration of NSSI during adolescence have been shown to significantly predict adult borderline personality disorder (BPD) (8). Also, several studies show NSSI to be a potent and unique risk factor for attempted suicide (11, 12). Young people engaging in NSSI are at increased risk for all forms of suicidal thoughts and behaviors, with as few as two to five episodes of NSSI conferring a fourfold increase in subsequent suicidal thoughts and behavior (13). Yet, only 17% of adolescents who engage in NSSI receive professional help for self-injury (2). NSSI is also not only associated with a variety of negative mental health outcomes, but can have profound consequences for others as well. Parents, for example, tend to feel overwhelmed and experience secondary mental distress after the discovery of their son's or daughter's NSSI (14). About one in three students also report that they know somebody who self-injures, which leads to stress among peers, and a desire for education and resource among teachers and school staff who frequently encounter youth who engage in NSSI [eg., (15)]. According to Hasking and colleagues (16), disclosure to parents, peers and teachers offers a positive outlook for future help-seeking, as long as the reaction to the NSSI disclosure occurs in an understanding and supportive manner. Research [eg., (17)] has shown that stigma related to NSSI as well as negative reactions (online/offline) increases the risk for NSSI and may create help-seeking barriers, and should, therefore, be targeted in the prevention of NSSI. Unfortunately, while there has been an increase in our understanding over the past decade of the factors that govern risk of NSSI [e.g., (18)], the development of evidence-based approaches for prevention remained nascent (18). In their review of NSSI prevention literature, primarily based on lessons learned from related health challenges, Heath et al. (19) and Kruzan & Whitlock (18) layout key considerations for evidence-based prevention programs targeting NSSI. The authors converge on the notion that school-based NSSI prevention and early intervention programs for young adolescents are likely to be most effective.

To the best of our knowledge, only one school-based prevention program for NSSI has been published, namely The Signs of Self-Injury (SOSI) program. The SOSI (20) program involves providing psychoeducation to school personnel (focusing on warning signs and response to NSSI disclosure) and developing guidelines for school policies. It also consists of a 50-min in-class-room component and focuses on teaching students to use the ACT model (Acknowledge the signs, Care for the person by showing desire to help, and Tell trusted adults) for supporting peers who self-injure. Results of a pilot study (21) showed no iatrogenic effects (i.e. no increase in NSSI thoughts and behaviors) and an increase in knowledge of NSSI, and greater comfort and perceived ability to assist peers who self-injure. While these findings are promising, Heath et al. point out that the study did not measure changes in help-seeking behavior of students currently engaging in NSSI or decreases in rates of NSSI thoughts/behaviors. Moreover, since the SOSI program focuses on providing school personnel and students with information and guidelines for responding to incidents of NSSI, this is a tertiary prevention program (i.e. intervening when the behavior has occurred to minimize further difficulties), rather than a primary (i.e. to prevent onset by intervening within a large normative population) or secondary (i.e. to delay the onset of the program by focusing on an at-risk group of individuals) prevention program. However, Heath et al. (19) stress the necessity of also developing evidence-based primary and secondary prevention strategies for NSSI. Several primary and secondary prevention programs focusing on mental health in general [e.g. Happyles and DBT in schools; (22–24)] and focusing on prevention of suicide in schools [e.g. the Saving and Empowering Young Lives in Europe (SEYLE) study; (25)] have been developed but the efficacy of these programs for NSSI is unclear.

The SEYLE (25) is a blended primary/secondary prevention program to prevent suicidality. In the SEYLE study, researchers examined the effectiveness of three prevention programs: gatekeeper training for school staff, screening for professionals, and Youth Aware of Mental Health program (YAM; five sessions of student role play, focusing on mental health). In this large-scale study, 11,110 adolescents (average age 15 years) were randomly allocated to prevention programs. Results showed a significant reduction in suicidal ideation and attempts for the YAM program but not for the gatekeeper training nor the screening program. Regrettably, effects on NSSI were not considered in this study.

DBT skills training for emotional problem solving for adolescents [DBT-A STEPS; (24)] is a school-based program for developing emotion regulation, interpersonal and problem-solving skills for middle and high school students (*secondary prevention*). While there are two evaluations of this program in educational settings, neither included NSSI measures (26, 27).

A promising new school-based *stepped-care prevention* program is Happyles [developed by Trimbos NL, (22)]. “Happyles” focuses on enhancing general mental well-being and social connectedness and is based on an eclectic approach grounded in positive psychology, cognitive behavioral therapy and problem solving. “Happyles” incorporates the following themes: fostering positive feelings, addressing negative thinking and stimulate positive thinking, taking control of one's life by managing problems or stress, becoming aware of future goals and making short-term plans to achieve them, and investing in connections with other people. Happyles has been shown to reduce internalizing symptoms, especially in high-risk groups (22, 23). However, the effectivity regarding the prevention of NSSI has not been examined thus far.

Although promising approaches exist, such as those described above, the effectiveness of school-based primary/secondary preventions programs with regard to prevention/delay in onset of NSSI is an important gap in the literature. In addition to the dearth of research, there is debate about whether or not prevention programs which focus on general mental health (and increased emotion regulation strategies and coping skills) are sufficient to prevent the onset of NSSI without specific components for addressing NSSI-related risk factors [e.g. (19)]. Some NSSI scholars who have considered this issue [e.g., (18, 28)] argue that effective school-based prevention will need to include NSSI-specific psychoeducation aimed at increasing awareness of NSSI as well as clear strategies for stopping the spread of contagion (and other NSSI-related factors such as NSSI stigma) in order to be effective to prevent/delay onset. However, most schools are afraid for potential iatrogenic effects. No empirical studies thus far have examined potential iatrogenic effects of a psychoeducational module on NSSI.

To this end, the first goal of this pilot study is to test the effectiveness of a school-based prevention program (i.e. Happyles) focusing on mental health for reducing NSSI behaviors in secundary school populations. Secondly, potential iatrogenic effects of a psychoeducational module on NSSI are examined. Furthermore, we will test whether adding a psychoeducation component targeting NSSI (NSSI awareness, decreasing stigma, the role of social media; HappylesPLUS) is beneficial in terms of a) NSSI-related outcomes (new onset, frequency, urges and liklihood of future engagement in NSSI), and b) secondary outcomes such as psychological distress (e.g., depressive symptoms), emotion regulation strategies, help-seeking, and de-stigmatization. Also, the experience of participants that followed the HappylesPLUS program will be explored using qualitative interviews.

## Methods

### Participants

The participants of this study were 651 pupils, between the ages of 11 and 15 (*M* = 12.85, *SD* =.769). In this sample, there were 323 (49.8%) girls, 326 (50.2%) boys and two pupils who did not indicate their sex (0.3%). Participants were recruited from six secondary schools in Belgium willing to participate in the study (i.e., convenience sampling). The researchers contacted the principals of some of the schools based on regional proximity, while other schools were known to the research unit because of previously reported NSSI incidents in these schools.

In total, 754 pupils were invited to participate in the study, of which 651 participated in both the pre- and post-measurements (response rate = 86.7%). Reasons for not participating were illness, conflicts in schedules of some pupils due to compulsory classes, one parent expressed concern about the potential iatrogenic effect of the program, and for some students it was too much of an intellectual challenge to fill out the questionnaires.

### Materials

All participants filled in demographic questions (about e.g., age and sex) and validated existing self-report questionnaires, including the Youth Outcome Questionnaire [YOQ-SR 30.1; (29)], the Brief Non-Suicidal Self-Injury Assessment Test [BNSSI-AT; (30)], the Difficulties in Emotion Regulation Scale [DERS; (31)], the Attitudes Toward Seeking Professional Psychological Help Scale—Short Form [ATSPPH-SF; (32)], and the Peer Mental Health Stigmatization Scale [PMHSS; (33)]. All questionnaires are administered during the first (t0) and the last happyles class (t1, approximately 6 weeks after T0).

The YOQ-SR 30.1 is a Dutch version of the YOQ (29), of which we used the self-report questionnaire. This questionnaire consists of 30 questions about the behavior of adolescents, scored on a 5-point Likert-type scale. The 30 items measure six subscales: Somatic Problems, Social Isolation, Aggression, Conduct Problems, Hyperactivity/Distractibility, and Depression/Anxiety). In this survey, the internal consistency of the total score is excellent: Cronbach's alpha is.90 at time 0 and.92 at time 1. The internal consistency of the subscales Conduct Problems (*α* =.75 at t0 and.79 at t1) and Depression/Anxiety (*α* =.74 at t0 and.77 at t1) and Aggression at time 1 (*α* =.70) is good. Finally, the internal consistency of the subscales Somatic (*α* =.66 at t0 and.69 at t1), Social Isolation (*α* =.67 at t0 and t1) and Hyperactivity/Distractibility (*α* =.65 at t0 and.63 at t1), and Aggression (*α* =.63 at t0) are acceptable.

Six items of the BNNSI-AT-NL, a Dutch translation of the Brief Non-Suicidal Self-Injury Assessment Tool (NSSI-AT), are used to screen NSSI (34). The BNNSI-AT-NL is a screening questionnaire for self-injuring behavior, developed by Whitlock and colleagues, and translated by Baetens and Claes (35). At t1 (at the end of the last Happyles class), students are asked to report their urge since the beginning of the Happyles-classes (t0), number of acts since the beginning of the Happyles-classes (t0), and likelihood for new acts in the future.

The Dutch translation of the DERS (31) measures two domains of difficulties in emotion regulation, namely “Lack of Emotional Awareness” and “Difficulties Controlling Impulsive Behavior when Experiencing Negative Emotions.” In our sample, the internal consistency of subscale “Lack of Emotional Awareness” is good (*α* at t0 =.81, *α* at t1 =.85). The internal consistency of “Difficulties Controlling Impulsive Behavior when Experiencing Negative Emotions” is also good at both time points (*α* at t0 =.85; *α* at t1 =.84).

The Dutch translation of the ATSPPH-SF (32) issued to get a better understanding of help-seeking behavior. The ATSPPH-SF is a self-report measure of attitudes toward seeking mental health care. The ATSPPH-SF measures two aspects of help-seeking behavior, namely openness to seeking treatment for emotional problems and value and need in seeking treatment (36). The ATSPPH-SF uses a 4-point Likert-type scale (0 = “Disagree” to 3 = “Agree”), whereas we use a 5-point scale. The short version has demonstrated good internal consistency in previous studies (*α* =.82–.84; 35). In the current study, the internal consistency for Openness is good (*α* at t0=.82 and *α* at t1 =.85). The internal consistency for value and need in seeking treatment, however, is unacceptable at both time 0 and time 1 (*α* =.37 at t0 and *α* =.46 at t1), and this subscale was therefore not used in the study.

Finally, in order to measure the change in the level of stigmatization toward adolescents with mental health problems, we utilize the Dutch translation of the PMHSS (33). The PMHSS contains 24 statements about peers with mental health problems that are rated on a 5-point Likert-type scale. In this study, we assess the subscales Stigma Agreement (personal endorsement of stigmatizing statements toward youth with mental health problems) and Stigma Awareness (awareness of prevailing societal stigma toward youth with mental health problems). McKeague et al. (33) showed that the PMHSS is a psychometrically sound instrument with good retest reliability. In our study, the internal consistency for Stigma Awareness and Stigma Agreement is good at time 0 (*α*=.70–.77) and at t1 (*α* =.83–.87).

### Procedure

This study was approved by the ethical committee of the university hospital of Brussels (Commissie Medische Ethiek UZ Brussel). All parents received a passive informed consent form *via* the schools, and all pupils signed an active informed consent form.

We randomly allocated the participating classes of 6 secundary schools to one of both conditions. We used Happyles as the basis of our universal prevention program for all participating pupils at all schools. Happyles is effective to enhance mental well-being in adolescents (22, 23), and is available in Dutch and is thus our primary choice as basis for a universal program (focusing on mental health, coping behaviors, emotion regulation, positive psychology). In total, 311 pupils (49.0% female) followed the regular Happyles program (henceforth referred to as Happyles). Each class received two in-classroom prevention lessons with classroom discussions and interactive assignments and two guided e-health lessons, all of which lasted about 50 min (see **Supplementary Material** for content of classes).

The other participants (n = 340; 50.4% female; henceforth referred to as HappylesPLUS), followed the Happyles program combined with the psychoeducation module on NSSI. This 50-min in-classroom module covered relevant topics in the prevention of NSSI [e.g., (17, 21)]: basic NSSI knowledge (prevalence, functions, risk factors), the role of social media, (de-)stigmatization of NSSI and help-seeking for NSSI. As no prevention module incorporating all of the above target themes for prevention was available in Dutch, Prof. Dr. Baetens developed the KRAS-module (including KRAS-documentary). This 50-min module started with a documentary in which young adults who used to engage in NSSI discussed what they experienced as being helpful. This documentary was followed by a guided class discussion on examples of self-care in general; advice for adolescents engaging in NSSI (to seek help), how to prevent NSSI contagion, and how to handle NSSI in social media. There also was a discussion on de-stigmatization of NSSI and how to help friends who engage in self-injury. The documentary and discussion often evoke emotional reactions among students (i.e., positive emotional exposure), so the KRAS-module ended with an emotion regulation exercise intended to soothe any overwhelming feelings. For more details on the content of the KRAS-module see **Supplementary Material**. This session was scheduled before the last Happyles class.

**Figure 1:**
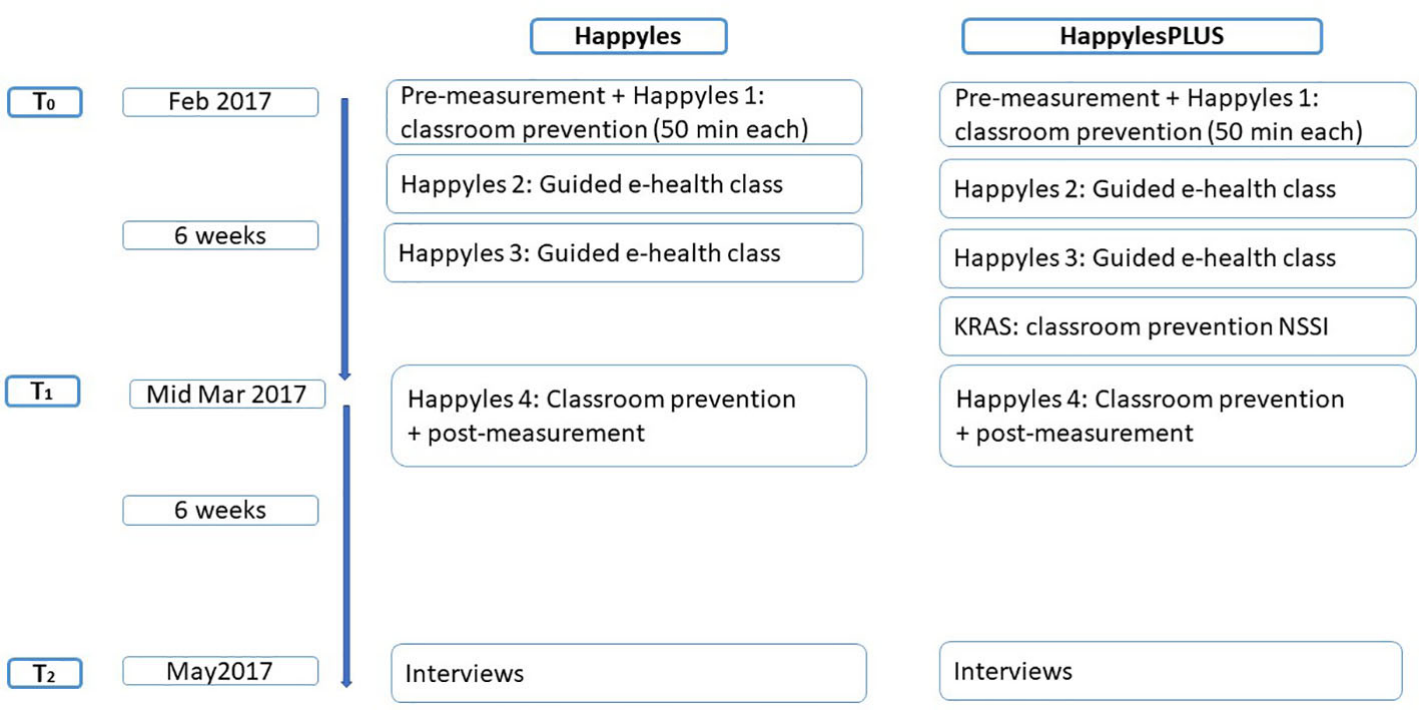
Diagram of the study procedures.

Six weeks after the four Happyles classes (and the KRAS-module), we organized a 15-min personal feedback session with all of the participants. These feedback sessions were an official part of the Happyles program. The first aim of these sessions was to refer the participants with an elevated risk profile to professional mental health care workers. The second aim of these individual sessions was to achieve a better understanding of how they perceived the intervention program *via* a semi-structured interview. These feedback sessions took place in the participating schools. See **Figure 1** for a diagram summarizing the study procedure.

### Analyses

Descriptive statistics are reported for the primary study variables as proportions (%) or mean values (M) with associated standard deviations (SD). The chi-square statistic was used to examine associations between categorical variables. Using paired t-tests, we examined mean change on continues scales between pre- and post-measurement across conditions. Linear regression analysis was used to evaluate sensitivity for change with mean change scores as dependent variables and baseline measurement, gender, age, and condition as independent variables. To allow for group and scale comparability, scores on all scales are expressed as percent of maximum possible scores (POMP) (37). Pomp scores rescale such that the score is the percentage of the distance from the minimum (0%) to the maximum (100%) of a scale. To examine whether ranks of ordinal data differ between pre- and post-measurement Wilcoxon signed-rank test was used, with differences in the rank difference score between conditions (i.e., Happyles vs. HappylesPLUS) tested with a Mann–Whitney U test. Alpha was consistently set to 0.05 and all analyses were performed using SPSS v. 23.

**Figure 2:**
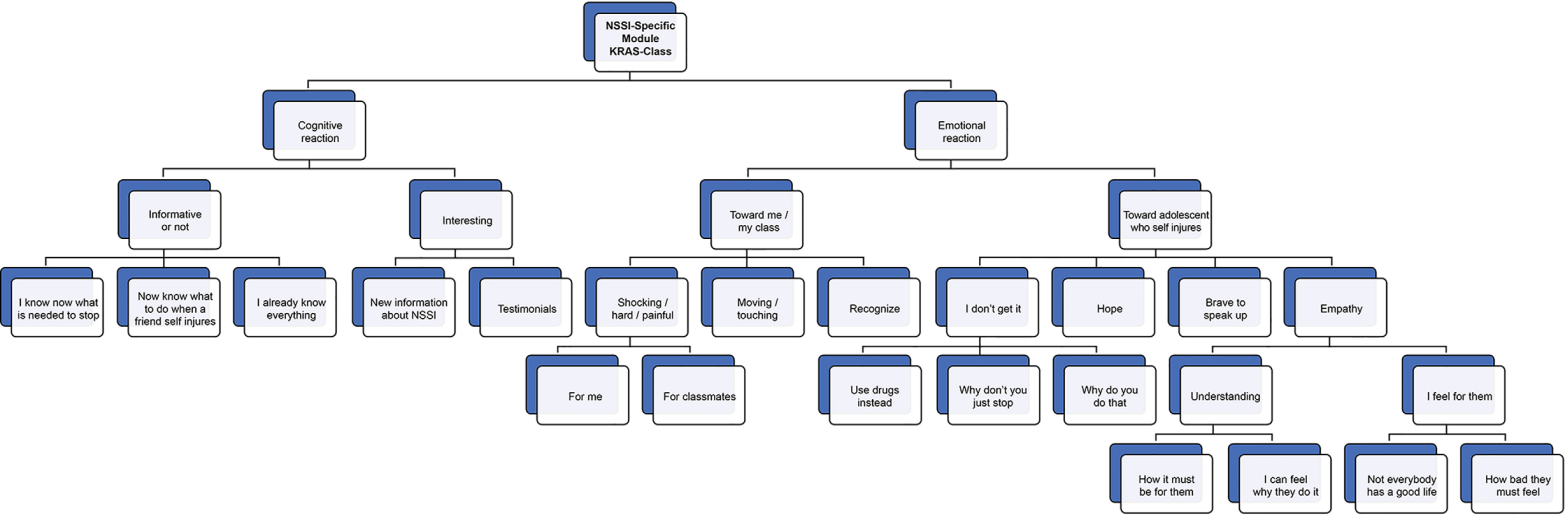
Hierarchical tree of qualitative experiences of NSSI-specific module. NSSI, non-suicidal self-injury.

The qualitative semi-structured interviews (of the HappylesPlus group only) were transcribed and ordered in an excel sheet. These data were analyzed with content analysis in MAXQDA software (38). It is a method for identifying, analyzing and reporting patterns (themes) within a data set as a means of identifying repeated patterns of meaning (39, 40). After the analysis, a hierarchical tree was created (see **Figure 2**).

## Results

### Preliminary Analyses

The data collected from the pre-measurement (t0) indicate that 14.9% of the sample reported a lifetime history of NSSI with a significantly, *χ*^2^(1) = 18.31, *p <*0.001, higher prevalence rate in girls (20.8%) than boys (8.8%). Of those with a history of NSSI, 49.4% reported having engaged in acts of NSSI for five days or more in their lifetime (6.7% of the sample). The prevalence of NSSI differed between both conditions, *χ*^2^(1) = 5.53, *p* = 0.019, with a higher percentage of students reporting a history of NSSI in the HappylesPLUS (18.1%) than the Happyles condition (11.4%). The three most commonly reported NSSI behaviors among those with a history of NSSI were cutting (44.1%), carving (30.1%), and wall/object punching (20.4%). The mean age of onset was 11.34 (SD = 2.14). Approximately one in four students (27.1%) reported at baseline significant levels of psychological distress (as determined by a cutoff of 29 or more on the Y-OC-Sr; Burlingame et al., 2001), with a significantly, (*χ*^2^(1) = 7.49, *p* = 0.006), higher proportion of students reporting elevated psychological distress in the HappylesPLUS (32.3%) than the Happyles condition (21.9%). Students with a lifetime history of NSSI were more likely to report elevated psychological distress (37.2% vs. 4.8%; *χ*^2^(1) = 94.87, *p* < 0. 001).

### Effectiveness With Respect to NSSI-Related Outcome Measures

Across the course of the study, 26 students reported new onset of NSSI (incidence rate of 6%). Incidence proportions did not differ significantly, *χ*^2^(1) = 1.27, *p* = 0.259, between the HappylesPLUS (4.7%) and the Happyles condition (7.3%). Of those with a history of NSSI at baseline, there were no significant differences between the HappylesPLUS (*M* = 3.58; *SD* = 6.52) and the Happyles condition (*M* = 2.11; *SD* = 4.93) in the number of days participants engaged in NSSI since the start of the program (*t*(70) = 1.01, p = 0.317). When asked about the urge to self-injure over the past month, adolescents who self-injure reported equally strong urges in the HappylesPLUS (*M* = 3.53; *SD* = 3.40) and the Happyles condition (*M* = 3.39; *SD* = 3.14). Interestingly, however, the result of a Wilcoxon Signed Rank Test revealed that students with a history of NSSI reported less perceived probability of future engagement in NSSI acts following both the HappylesPLUS condition, (*Z*(42)= 6.09, p < 0.001) and the Happyles condition (*Z*(27) = 4.33, p < 0.001). This rank-order effect appeared not to differ significantly between both conditions (*U*= 509, *p* = 0.458).

### Effectiveness With Respect to Secondary Outcome Measures

When considering changes on the YOQ 30.1, we observed no significant changes across both conditions on the Total Psychological Distress score, or for the subscales Social Isolation, Aggression, Hyperactivity/Distractibility, and Depression, and Anxiety symptoms (see **Table 1**). In both conditions, however, students reported a decrease in Somatic Problems. There was also an increase in Conduct Problems from pre- to post-measurement in the HappylesPLUS condition. A similar trend was apparent in the Happyles condition, but this did not reach statistical significance (*p* = 0.069; **Table 1**).

**Table 1:** Changes in psychological distress from pre- to post-measurement.

	Pre- measurement	Post-measurement	Mean-difference (post-pre)		*p*
	M (SD)	M (SD)	M (SD)	95% CI	
Total psychological distress					
Happyles	18.01 (10.23)	18.18 (11.89)	0.18 (9.49)	−1.06; 1.42	0.780
HappylesPLUS	18.54 (11.94)	18.94 (13.5)	0.41 (9.11)	−0.75; 1.57	0.489
Somatic symptoms					
Happyles	25.39 (19.36)	22.72 (18.61)	−2.67 (19.42)	−4.96; −0.37	0.023
HappylesPLUS	28.13 (22.13)	26.23 (23.62)	−1.90 (16.06)	−3.73; −0.07	0.042
Social isolation					
Happyles	12.81 (20.61)	12.14 (19.16)	−0.67 (18.39)	−2.83; 1.49	0.543
HappylesPLUS	11.65 (18.99)	12.42 (19.59)	0.76 (17.17)	−1.21; 2.73	0.446
Aggression					
Happyles	5.73 (10.95)	6.76 (12.55)	1.03 (13.2)	−0.54; 2.61	0.198
HappylesPLUS	7.28 (13.48)	8.08 (13.92)	0.80 (13.08)	−0.71; 2.67	0.298
Conduct problems					
Happyles	13.16 (11.86)	14.44 (13.59)	1.28 (11.48)	−0.10; 2.67	0.069
HappylesPLUS	14.93 (14.31)	16.62 (16.11)	1.69 (12.79)	0.18; 3.19	0.028
Hyperactivity/Distractibility					
Happyles	35.35 (23.41)	34.84 (22.21)	−0.51 (20.24)	−2.91; 1.88	0.674
HappylesPLUS	32.88 (20.33)	33.22 (20.12)	0.37 (17.66)	−1.67; 2.42	0.719
Depression/Anxiety					
Happyles	21.51 (16.13)	20.85 (16.0)	−0.67 (14.39)	−2.41; 1.08	0.454
HappylesPLUS	22.07 (17.30)	21.75 (18.0)	−0.33 (13.2)	−1.89; 1.25	0.689

M, mean; SD, standard deviation; CI, confidence interval; p, probability. Paired t-tests examining change from pre- to post-measurement per program.

Importantly, however, mean change scores did not differ significantly between conditions on any of the scales of the YOQ 30.1 (**Table 2**). Students with higher scores at baseline were more likely to show a decrease from pre- to post-measurement in both groups. Boys showed a significantly stronger reduction than girls on the Somatic Complaints scale (mean difference = 3.83; SE = 1.38, *p* = 0.006), while girls showed a stronger reduction than boys on the Aggression scale (mean difference = 2.73; SE = 0.98, *p* = 0.006). Older adolescents were also less likely to report a reduction on the Aggression scale.

**Table 2:** Linear regression predicting change from pre- to post-measurement in psychological distress scales.

	B	SE	T	*p*
Total psychological distress				
Score pre-measurement	−0.19	0.04	4.96	<0.001
Condition (HappylesPlus)	0.16	0.85	0.18	0.855
Gender (girl)	0.88	0.85	1.04	0.299
Age	0.44	0.61	0.72	0.473
Somatic symptoms				
Score pre-measurement	−0.35	0.03	10.56	<0.001
Condition (HappylesPlus)	1.59	1.39	1.15	0.251
Gender (girl)	3.83	1.38	2.78	0.006
Age	0.93	0.96	0 .97	0.334
Social isolation				
Score pre-measurement	−0.43	0.03	12.6	<0.001
Condition (HappylesPlus)	1.04	1.35	0.77	0.444
Gender (girl)	1.8	1.35	1.34	0.182
Age	0.02	0.92	0.02	0.987
Aggression				
Score pre-measurement	−0.50	0.04	11.96	<0.001
Condition (HappylesPlus)	−0.11	0.99	0.11	0.915
Gender (girl)	−2.73	0.98	2.78	0.006
Age	1.6	0.68	2.37	0.018
Conduct problems				
Score pre-measurement	−0.28	0.04	7.13	<0.001
Condition (HappylesPlus)	0.38	1.02	0.38	0.707
Gender (girl)	0.78	1.01	0.78	0.439
Age	0.97	0.71	1.37	0.17
Hyperactivity/Distractibility				
Score pre-measurement	−0.41	0.03	12.68	<0.001
Condition (HappylesPlus)	−0.41	1.44	0.29	0.773
Gender (girl)	0.96	1.42	0.67	0.501
Age	1.89	0.98	1.93	0.055
Depression/Anxiety				
Score pre-measurement	−0.32	0.03	9.22	<0.001
Condition (HappylesPlus)	0.41	1.11	0.37	0.713
Gender (girl)	1.13	1.14	1	0.32
Age	0.67	0.77	0.87	0.382

Linear regression analyses with mean change scores (t1–t0) as dependent variables and baseline measurement, gender, age, and condition as independent variables to evaluate sensitivity for change.

Next, we evaluated changes in emotion regulation regarding Impulse Control Difficulties and Lack of Emotional Awareness (**Table 3**). Although no change was observed regarding Impulse Control Difficulties across both conditions, we observed a significant improvement in Awareness of Emotions from pre- to post-measurement across both conditions. Again, mean change scores did not differ significantly between conditions on any of emotion regulation scales (**Table 4**), although there was a trend indicating a greater improvement in emotional awareness in the HappylesPLUS condition (p = 0.087) relative to the Happyles condition. Older students and those with higher scores at baseline showed greater improvement following the prevention programs on both the Impulse Control Difficulties and Lack of Emotional Awareness scale.

**Table 3:** Changes in emotion regulation from pre- to post measurement.

	Pre-measurement	Post-measurement		Mean-difference (post-pre)		*p*
	M (SD)	M (SD)		M (SD)	95% CI	
Lack of awareness of emotions						
Happyles	48.01 (22.33)	42.78 (23.82)		−5.23(24.51)	−8.21; −2.25	0.001
HappylesPLUS	44.07 (22.91)	36.70 (23.51)		−7.37 (24.65)	−10.29; −4.45	<0.001
Impulse control difficulties						
Happyles	74.03 (23.05)	74.70 (22.57)		0.67 (21.97)	−2.04; 3.39	0.626
HappylesPLUS	71.88 (23.97)	73.75 (21.81)		1.88 (22.52)	−0.85; 4.61	0.177

M, mean; SD, standard deviation; CI, confidence interval; p, probability. Paired t-tests examining change from pre- to post-measurement per program.

**Table 4:** Linear regression predicting change from pre to post-measurement in emotion regulation.

	B	SE	T	*p*
Lack of awareness of emotion				
Score pre-measurement	−0.54	0.04	13.09	<0.001
Condition (HappylesPlus)	−3.21	1.88	1.71	0.087
Gender (girl)	1.32	1.87	0.71	0.479
Age	−2.67	1.29	2.07	0.039
Impulse control difficulties				
Score pre-measurement	−0.50	0.04	13.89	<0.001
Condition (HappylesPlus)	0.88	1.69	0.52	0.602
Gender (girl)	−0.51	1.67	0.31	0.759
Age	−3.22	1.16	2.79	0.006

Linear regression analyses with mean change scores (t1–t0) as dependent variables and baseline measurement, gender, age, and condition as independent variables to evaluate sensitivity for change.

With respect to Openness to Seeking Treatment for Emotional Problems, neither the HappylesPLUS, (Mt0 = 43.04 SD = 23.69, Mt1 = 43.45 SD = 24.44, 95% CI mean difference = −2.15; 2.97, *p* = 0.751), nor the Happyles condition (Mt0 = 47.23 SD = 21.42 Mt1 = 47.78 SD = 21.00, 95% CI mean difference = −1.89; 2.98, *p* = 0.657) showed any significant change over time. Condition, gender, and age did not significantly predict mean change from pre- to post-measurement (all *p* > 0.30). Finally, we evaluated change in the level of stigmatization toward adolescents with mental health problems. Again, we did not find any significant change in Stigma Awareness and Stigma Agreement across both conditions (**Table 5**). Condition and age did not significantly predict mean change from pre- to post-measurement in Stigma Awareness and Stigma Agreement (**Table 6**). Students with higher stigma scores at baseline, however, showed greater improvements on both scales, and female students reported greater improvements in Stigma Agreement than their male peers following the prevention programs.

**Table 5:** Changes in stigmatization of mental health problems from pre- to post-measurement.

	Pre-assessment	Post-assessment	Mean-difference (post-pre)		*p*
	M (SD)	M (SD)	M (SD)	95% CI	
Stigma awareness					
Happyles	41.51 (16.70)	41.19 (19.02)	−0.32 (20.06)	−2.77; 2.14	0.801
HappylesPLUS	42.09 (16.32)	41.08 (18.12)	−1.01 (17.74)	−3.11; 1.09	0.346
Stigma agreement					
Happyles	30.53 (15.46)	31.22 (16.09)	0.69 (16.47)	−1.39; 2.77	0.512
HappylesPLUS	32.49 (14.82)	32.99 (16.82)	0.51 (17.47)	−1.56; 2.57	0.630

M, mean; SD, standard deviation; CI, confidence interval; p, probability. Paired t-tests examining change from pre- to post-measurement per program.

**Table 6:** Linear regression predicting change from pre to post-measurement in stigmatization of mental health problem.

	B	SE	T	*p*
Stigma awareness				
Score pre-measurement	−0.51	0.05	11.48	<0.001
Condition (HappylesPlus)	−0.32	1.49	0.21	0.833
Gender (girl)	−1.57	1.47	1.07	0.285
Age	0.76	1.02	0.74	0.457
Stigma agreement				
Score pre-measurement	−0.55	0.04	12.47	<0.001
Condition (HappylesPlus)	0.68	1.33	0.51	0.612
Gender (girl)	−3.41	1.32	2.59	0.01
Age	1.36	0.89	1.52	0.13

Linear regression analyses with mean change scores (t1–t0) as dependent variables and baseline measurement, gender, age, and condition as independent variables to evaluate sensitivity for change.

### Qualitative Experiences of Pupils With Regard to the NSSI-Module

**Figure 2** provides an overview of the main experiences of the pupils (in the HappylesPlus group) with regard to the NSSI-specific module, based on the content analyses of the qualitative interviews. There were two main theme categories: cognitive reactions and emotional reactions.

The participants whose reactions were classified as cognitive often used the words “interesting,” “informative,” “knowing,” and “knowledge.” Participants thought the lessons were interesting because they provided them with new information on NSSI (e.g., what it is about, how it differs from suicidal behavior, how NSSI might become problematic, and reasons why adolescents engage in self-injury). Some explicitly mentioned that they appreciated the documentary because of the testimonials (which made it real). Furthermore, the lessons were thought to be informative because they provided them with tools to help a friend/classmate who is engaging in NSSI (organize fun activities, show the person that you are there for him/her, tell them they should not be embarrassed, ask the person questions about his behavior, and be discrete about the information you get). In this regard, one advice was quoted a lot: “convince the other to ask for help.” Some students stated that they now know better what is needed to recover from self-injury, whereas others stated that they already knew everything that was covered in the NSSI-focused session.

The emotional reactions concerning the KRAS-class are divided between how pupils feel about the class (for themselves and/or their classmates) and how they feel about adolescents who self-injure in general. Some pupils perceived the KRAS-class as shocking and painful: They imagined that it can be difficult for classmates who are engaging in NSSI (or have engaged in NSSI). One pupil indicated that she left the classroom as she found it too difficult to watch the documentary and indicated that people shouldn't do such a thing. A few pupils mentioned that the documentary was moving or touching. Some pupils mentioned that the documentary motivated them to be positive toward life (e.g., even when life may seem bad, there are always good things to focus on). Some emotional reactions are related to adolescents who self-injure (or adolescents who testified in the documentary): “I feel with them,” “I know now what they must go through,” “I will not judge someone who is self-injuring anymore,” and “I can see now that not everyone has the same, good life.” There were, however, some students who continued to demonstrate shock and disapproval toward individuals who self-injure: “Why would you do such a thing? They just want negative attention.” Finally, some participants focused on hope for people engaging in NSSI: “you always have a chance to overcome it.”

### Experiences of Those With Lived Experience Regarding the NSSI-Specific Module

Students with lived experience were asked how they perceived the HappylesPLUS program and which elements they considered relevant. Most of them explained that they now realize how important it is to talk to others about their feelings. A majority of participants with a history of NSSI also explicitly mentioned they now know where to seek help, and most of them indicated they are planning to go into therapy or have already started therapy. Some of them stated that they have a better insight into which strategies might be helpful to them to decrease the urge to engage in NSSI. A few pupils who engage/have engaged in NSSI also disclosed that they experienced the KRAS-class as confronting. Others indicated it is reassuring to know they are not the only ones struggling with NSSI. Finally, some mentioned that they now feel more supported by their classmates, and reported that the KRAS-class gave them hope for the future.

## Discussion

This pilot study examined the effectiveness, and feasibility, of combining a school-based prevention program (i.e. Happyles) focusing on mental health with a psychoeducation module on NSSI.

The lifetime prevalence rate of 14.9% in this sample of Flemish pupils is higher than expected at this age (e.g. prevalence of 8% in the age group of 11 to 14 years) (41). This is likely due to the fact that some schools which had previously encountered problems with NSSI in their schools were highly motivated to participate in the study. The higher rates among girls in early adolescence is consistent with previous studies (5, 40). In line with earlier findings (e.g., (28)], the present study confirms that there is a strong relationship between NSSI and higher levels of psychological distress.

With regard to the primary outcome, we found evidence that both Happyles and HappylesPLUS show a significant decrease in the probability of future engagement of NSSI. In line with the results of the SOSI study (21), results revealed no iatrogenic effects as the incidence rates and frequency of NSSI did not differ between Happyles and HappylesPLUS. While this is reassuring, we did not find lower incidence rates or reduced NSSI frequency directly after the 4-week program of the HappylesPLUS. Future work is needed to evaluate the potential longer-term benefits of Happyles(PLUS) in the delay of onset/frequency of NSSI. Regarding secondary outcomes, we observed mainly a decrease in somatic complaints and an increase in conduct problems. The latter is likely to be connected to the increase of emotional awareness as a result of the program. All results show a floor-effect in line with previous studies (22, 23), indicating that especially pupils who show an elevated level of psychological complaints benefit from the prevention. Furthermore, although we did not observe a measurable impact on help-seeking for mental well/ill-being, the qualitative interviews revealed that the NSSI-specific module for adolescents who are engaging in NSSI may be beneficial as some students reported increased motivation to seek professional help for NSSI (and talk to peers about their emotions). Similarly, after the NSSI-specific psychoeducation module students without a history of NSSI indicated during the semi-structured interviews that they learned that it is important to motivate peers who self-injure to seek professional help. Regarding stigma, results show no overall change across both groups in the self-reported questionnaires. The qualitative data show a decrease in NSSI-stigma for some students. Due to the discrepancy between the questionnaires (examining for example stigma or help-seeking for psychopathology in general) and the qualitative data, we advice future studies to examine changes in stigma and help-seeking specifically for NSSI using questionnaires that are sufficiently sensitive to change.

Overall, we conclude that a general school-based prevention program may have a positive effect on the likelihood of future engagement in NSSI. Adding an NSSI-psycho-educational module to this general mental health prevention did not show any iatrogenic effects and may have benefits to adolescents who are at high risk or who are already engaging in NSSI. Recent developments in NSSI research, as for example Kiekens and colleagues (42) demonstrated, make it possible to detect individuals at high risk for beginning self-injury with reasonable accuracy. An important avenue for future research will be to evaluate which type of interventions work best for adolescents at varying levels of risk.

While this pilot study takes an important step in showing the feasibility of preventive interventions for NSSI, the results need to be interpreted with several important limitations in mind. First, since we do not have long-term follow-up data, empirical investigation of long-term effects is lacking. Second, cultural differences should be taken into account. Happyles uses quotes of and movies with Dutch adolescents: we noticed that our Flemish pupils did not always fully grasp all content (due to cultural differences). Third, we cannot account for the significant difference in prevalence of NSSI between the Happyles and HappylesPLUS group: we wonder if the conditions were not blind enough for the administrative personnel of the schools as they had to plan four or five classes. Furthermore, we noticed substantial diversity in the participating schools, not just with regard to the prevalence of engagement in NSSI behavior, but also with regard to school climate and stigmatization of psychological symptoms. Unfortunately, we did not include the school climate in our questionnaires. Future studies might take into account school climate as a factor in the effectiveness of a school-based prevention program targeting NSSI. Fourth, this study included a brief intervention period between the four or five classroom hours (between pre-and post-measurement there were 6 weeks maximum), limiting the number of possible new onsets of NSSI. Finally, we did not examine the effect of the personal feedback sessions. However, based on the appraisals of students (and actual referrals to professional health care), we encourage researchers to consider this when developing an evidence-based school-based prevention program targeting NSSI.

## Conclusion

This pilot study shows that incorporating NSSI-specific modules to evidence-based school prevention programs is feasible and does not lead to iatrogenic effects. Another important finding from this study is that we observed reduced likelihood of future engagement in NSSI following a general school-based prevention program (with and without NSSI module). Intriguingly, qualitative interviews indicate that the addition of an NSSI-specific module may have direct benefits to students with lived experience: as some mentioned they now realize that it is important to talk to others about their feelings and are motivated to seek professional help. This study underlines the importance of future research developing an evidence-based program for preventing NSSI. Targeting the onset of NSSI in secondary school may provide a brief window of opportunity to mitigate the risk of developing NSSI.

## Data Availability Statement

The datasets generated for this study are available on request to the corresponding author.

## Ethics Statement

The studies involving human participants were reviewed and approved by Ethische commissie UZBrussel, Vrije Universiteit Brussel. Written informed consent to participate in this study was provided by the adolescents and the participants' legal guardian/next of kin.

## Author Contributions

IB contributed to the conception and design of this study as well as analyses/interpretation of results. She took the lead in writing of the manuscript. CD contributed to the conception and design of this study, and revised the manuscript critically. SV assisted in the interpretation of results and revised the manuscript critically. BV analyzed and interpreted the qualitative data and took the lead in the results section with regard to the qualitative data. GK revised the manuscript critically and took the lead in the quantitative data-analyses and interpretation of results. All authors gave final approval and agree to be accountable for all aspects of the work ensuring integrity and accuracy.

## Conflict of Interest

The authors declare that the research was conducted in the absence of any commercial or financial relationships that could be construed as a potential conflict of interest.
