# Supplementary material for: School-Based Prevention Targeting Non-Suicidal Self-injury: A Pilot Study
Source: Front Psychiatry. 2020 May 29;11:437. doi: 10.3389/fpsyt.2020.00437 (PMC7298560; doi:10.3389/fpsyt.2020.00437)
Supplement: Supplementary file 1 [file Table_1.docx]

SUPPLEMENTARY MATERIAL

**Content classes HAPPYLES(PLUS)**

| **Session** | **Topics** | **Examples of excercises / activities** |
| --- | --- | --- |
| 1. Pre-measurement and Happyles introduction class | General mental health | Opinion game, speeddate game, quiz |
| 1. Guided e-health lesson | Guided e-health lesson about fostering positive feelings and the interplay between thoughts, feelings and behaviour | - name 5 things that make you happy  - quiz about happiness  - life consists of various domains + each domain of life can be positive or less positive  - which activities give you an instant feeling of happiness  - events you can vs events you cannot influence  - your thoughts about events influence your mood  - how to change negative thoughts  - helping vs non-helping thoughts and their influence on mood and behavior  - opinion game  - constructive communication  - do something nice for someone else |
| 1. Guided e-health lesson 2 | Guided e-health lesson about problem solving,  positive relations with other people &  future goals | - important vs unimportant problems and problems you can solve vs problems you cannot solve  - first aid in case of stress (chill-out exercises, talking to someone you trust, doing fun things)  - your goals for the near/ middle/long term future  - what are your talents  - advice on how to stop ruminating |
| 1. HAPPYLESPLUS condition only: KRAS class (developed by Prof. Dr. Baetens) | NSSI awareness, decrease stigma, encourage help seeking, taking care of self-and others, and dealing (with NSSI) in social media | -Introduction  -Documentary (focussing on hope, support, but also an emotional exposure to positive emotions)  -Guided discussion  -provide information and addresses for referral/information on NSSI  - relaxation exercise to end the class to cope with potential overwhelming feelings. |
| 1. Final class and post-measurement | Cognition & emotion | -explanation on cognitive distortions  - exercises on cognitive distortions |
| 6.Individual feedback session | Each pupil was invited for a 15-minute individual talk (to avoid stigma).  At risk students (e.g., high rates of depression, suicidal thoughts, NSSI) were referred to professional health care. If students gave their permission, we called their parents during this talk. | There scores on the ‘happyles – test’ and relevant indicators were discussed, and we asked each of them how they were feeling, and if they have somebody to talk to.  Brief semi-structured interview on their experiences |
